# Living with Metastatic Cancer: A Roadmap for Future Research

**DOI:** 10.3390/cancers12123684

**Published:** 2020-12-08

**Authors:** Danielle B. Tometich, Kelly A. Hyland, Hatem Soliman, Heather S. L. Jim, Laura Oswald

**Affiliations:** 1 Department of Health Outcomes and Behavior, Moffitt Cancer Center, Tampa, FL 33612, USA; danielle.tometich@moffitt.org (D.B.T.); Heather.Jim@moffitt.org (H.S.L.J.); 2 Department of Psychology, University of South Florida, Tampa, FL 33612, USA; khyland@usf.edu; 3 Department of Breast Oncology, Moffitt Cancer Center, Tampa, FL 33612, USA; hatem.soliman@moffitt.org

**Keywords:** metastatic cancer, quality of life, symptoms, symptom management, distress, melanoma, breast cancer, survivorship

## Abstract

**Simple Summary:**

Although people with metastatic cancer are living longer with their disease, they are faced with challenges that can interfere with their quality of life. These challenges include worsening disease, survival time, symptoms, distress, and financial problems. The aim of this review paper is to describe a framework to guide future efforts to address these challenges. The framework includes the risk factors (predisposing factors), triggers (precipitating factors), and responses (perpetuating factors) that contribute to the onset and maintenance of problems in living with metastatic cancer. We conclude by suggesting areas for future work to prevent problems, manage triggers, and reduce unhelpful responses.

**Abstract:**

Living with metastatic cancer, or metavivorship, differs from cancer survivorship and has changed as novel treatments have increased survival time. The purpose of this narrative review is to describe factors that impact challenges in metavivorship within a conceptual framework to guide future research. This review focuses on the specific metavivorship outcomes of progressive disease, survival time, symptoms, distress, financial toxicity, and quality of life. We describe the predisposing, precipitating, and perpetuating (3P) model of metavivorship. Understanding the biological, psychological, and social 3P factors that contribute to the development and maintenance of challenges in metavivorship provides a roadmap for future research. Implications of this model include prevention by targeting predisposing factors, management of precipitating factors after onset of metastatic disease, and treatment of perpetuating factors to reduce symptoms and improve quality of life during the chronic phase of metavivorship. This can be accomplished through biopsychosocial screening efforts, monitoring of patient-reported outcomes, education and communication interventions, interdisciplinary symptom management, advance care planning, and behavioral interventions to cultivate psychological resilience.

## 1. Introduction

In 2006, the Institute of Medicine published a seminal report entitled “From Cancer Patient to Cancer Survivor: Lost in Transition” that described the challenges of transitioning from active cancer treatment to post-treatment survivorship [1]. Its emphasis on high quality survivorship care is as relevant today as it was when the report was published. However, a new group of people with cancer has since emerged: those living long-term with metastatic disease. Advances in immunotherapy and targeted therapies have led to significant improvements in survival outcomes among patients with a variety of recurrent or de novo metastatic cancers. For these patients, the commonly used definition of a cancer “survivor” as a person who is disease-free after curative treatment does not apply. Instead, patients living with metastatic disease increasingly refer to themselves as “metavivors” or people living with cancer as a chronic and terminal illness [2]. Metavivors face unique challenges, including an uncertain prognosis, management of acute and chronic symptoms, financial burden, and the need for caregiving by family and friends [3,4]. However, research on these issues in metavivors has been extremely limited [5,6].

The goal of the current review is to describe a conceptual framework and roadmap for future research on metavivorship. We focus on metastatic breast cancer and melanoma as examples due to the large number of metavivors with these diagnoses. However, therapeutic advances have also resulted in extended survival for several other advanced cancers including renal cell carcinoma, hepatocellular carcinoma, colorectal, gastric, and head and neck cancers [7,8]. More research is needed on metavivorship across all of these diagnoses. We also focus on CDK4/6 inhibitors and immune checkpoint inhibitors in metastatic breast cancer and melanoma, respectively, as large numbers of metavivors with these diagnoses are treated with these agents. However, metavivors are treated with a wide variety of targeted therapies and their experiences should also be studied.

The review begins with an overview of the predisposing, precipitating, and perpetuating (3P) Model as a framework for biopsychosocial research on metavivorship and identifies existing evidence and areas for future research within each part of the 3P Model. Although the 3P Model can be broadly applied to a variety of outcomes, including disease progression and survival, the current review focuses primarily on the experience of metavivorship as described through patient-reported outcomes (PROs). PROs are “any report of the status of a patient’s health condition that comes directly from the patient, without interpretation of the patient’s response by a clinician or anyone else” [9], p. 2. PROs include disease symptomatology, side effects of treatment, and quality of life (i.e., the impact of symptoms and side effects on daily functioning). Opportunities are discussed for interdisciplinary interventions to improve PROs, and the review concludes with a discussion of the potential impact of metavivorship research on patient outcomes and quality of care.

## 2. The Predisposing, Precipitating, and Perpetuating (3P) Model of Metavivorship

**Figure 1:**
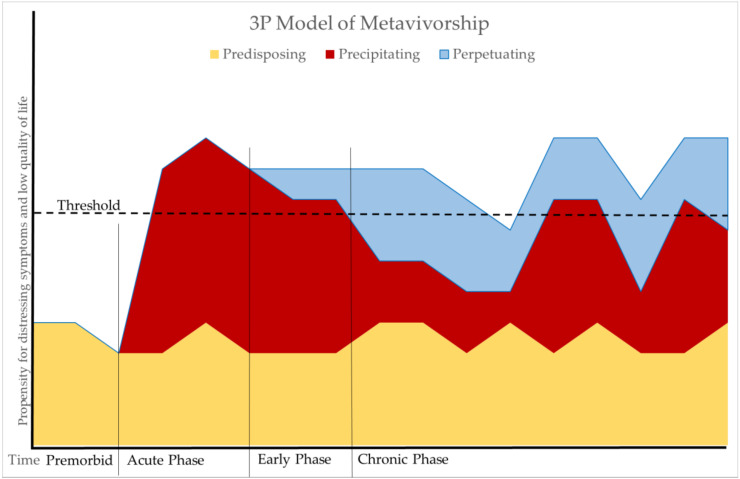
The predisposing, precipitating, and perpetuating (3P) Model of Metavivorship. Predisposing factors may vary over time in their contribution to distressing symptoms and low quality of life. In the premorbid phase prior to diagnosis of metastatic or recurrent disease, predisposing factors are the sole contributor to risk for developing distressing symptoms. Precipitating factors begin in the acute phase, begin to decline in the early phase, but may vary throughout the chronic phase due to various treatments and disease monitoring. Perpetuating factors begin in the early phase and can sustain symptoms and affect quality of life throughout the chronic phase.

**Figure 2:**
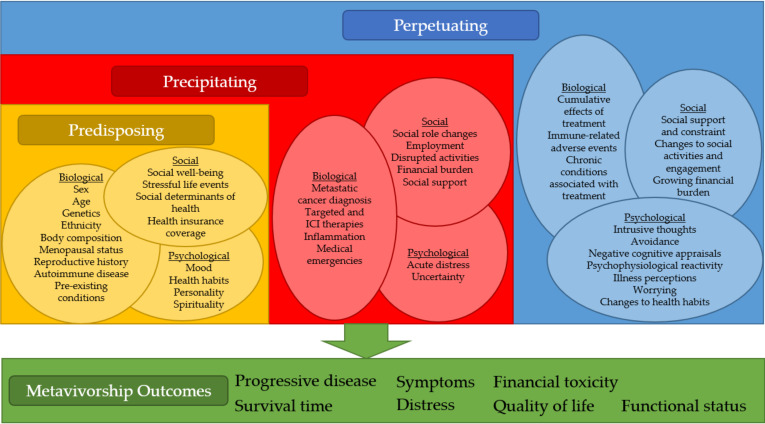
Biopsychosocial Predisposing, Precipitating, and Perpetuating (3P) Factors and Metavivorship Outcomes.

The 3P Model provides a basis for understanding how predisposing, precipitating, and perpetuating factors contribute to the development and maintenance of long-term conditions [10,11]. Due to its focus on the dynamic interplay of these factors over time, it is an ideal model with which to conceptualize metavivorship (Figure 1). As it relates to metavivorship, the model posits that predisposing factors exist prior to a diagnosis of metastatic disease. Biological, sociodemographic, clinical, and behavioral predisposing factors may contribute to the development of metastatic disease as well as adverse outcomes such as reduced survival, greater symptomatology, and worse quality of life. Precipitating factors are caused by disease and treatment, and include biological, psychological, and behavioral factors. They may be transient, such as distress about one’s diagnosis or acute side effects of treatment; sustained, such as inflammatory processes from cancer or its treatment; or recurrent, such as adverse events resulting from different sequences of therapies. Perpetuating factors are longer-term biological, psychological, behavioral, and social changes due to precipitating factors that can sustain or exacerbate poor outcomes. Examples of biopsychosocial 3P factors and metavivorship outcomes are shown in Figure 2.

The 3P Model suggests that interventions can be implemented at each phase of illness. Targeting individuals at risk due to predisposing factors may prevent onset of metastatic disease or side effects of treatment. Following a diagnosis of metastatic disease and initiation of precipitating factors, effective management of precipitating factors may prevent the development of perpetuating factors. Finally, perpetuating factors may be addressed in the chronic phase with appropriate intervention. Identifying the predisposing, precipitating, and perpetuating factors that contribute to outcomes can inform treatment planning and decision making at each stage of metavivorship.

### 2.1. Predisposing Factors

Biological, clinical, and behavioral predisposing factors for a diagnosis of metastatic breast cancer or melanoma are well known. For breast cancer, they include such factors as BRCA1/2 mutations, family history of breast cancer, reproductive history, breast density, hormone replacement therapy, physical inactivity, overweight/obesity, and alcohol use [12,13,14,15]. For melanoma, they include indoor tanning, sun exposure, genetic variation (e.g., MC1R, CDKN2A), family history, dysplastic nevi, and immunosuppression (e.g., solid organ transplant, cyclosporine, sirolimus) [16]. Across cancer types, genetic variation and interactions between genetics and environmental and behavioral factors appear to play an important role in susceptibility for metastatic cancer (see [17] for a more extensive review of genetic and ethnic risk factors). Less data are available regarding predisposing factors for treatment efficacy. Although direct head-to-head comparisons have not been conducted to our knowledge, secondary analyses from clinical trials suggest that CDK4/6 inhibitors (i.e., palbociclib, ribociclib, abemaciclib) improve survival in metastatic breast cancer regardless of age, histological type, history of prior neoadjuvant/adjuvant systemic treatment, or sites of distant metastasis. Interestingly, there are data to suggest that abemaciclib combined with aromatase inhibitors is more efficacious in normal or underweight patients compared to overweight or obese patients [18]. For melanoma, data suggest no differences in the efficacy of immune checkpoint inhibitors (ICIs; i.e., ipilimumab, nivolumab, pembrolizumab) by age, comorbidity, income quintile, or previous interferon treatment [19]. There are mixed data to suggest that men may benefit more from ICIs than women [19,20,21]. Overweight and obese melanoma patients have better response rates to ICIs as well as better overall survival and progression-free survival [22,23,24,25]; however, the mechanisms for these survival advantages are not clear. Notably, access to ICIs is not equitable. According to data from National Cancer Database, ICIs were more likely to be administered to younger and healthier patients and those receiving treatment at academic medical centers [26]. African Americans and patients with Medicaid and lower incomes were less likely to receive ICIs [26]. Similarly, a comparison of melanoma survival rates using SEER data from pre- to post-FDA approval of ipilimumab indicated dramatic increases in survival among patients with insurance and those from urban or low-poverty areas [27]. In contrast, there were no survival improvements for patients who were uninsured or from rural or high-poverty areas.

Few studies have examined predisposing factors for PROs such as patient-reported symptomatology and quality of life in the context of metavivorship. Notably, because side effects of targeted therapies and ICIs are significantly different than those of standard chemotherapy, previous research on PROs among metastatic patients is of limited utility. Data from metavivors come primarily from subgroup analyses of adverse events (AEs) reported on clinical trials. Predisposing factors for PROs represent a significant knowledge gap, as it is well-established that clinician-rated AEs underestimate the frequency and severity of patient-reported symptoms by as much as 50% [28,29,30,31]. Nevertheless, available data regarding subgroup differences in AEs suggest potential avenues for PRO research.

Regarding CDK4/6 inhibitors for metastatic breast cancer, AEs and dose reductions/interruptions are common. Neutropenia, the most common AE due to palbociclib or ribociclib, occurs in up to 75% of patients, with up to 48% experiencing grade 3–4 neutropenia [32,33]. Interestingly, neutropenia appears to be more common in Asian patients [34] and those over the age of 75 [35]. In contrast, abemaciclib, which binds more selectively to CDK4 and has a different AE profile, is commonly associated with any grade of diarrhea (up to 81%), neutropenia (up to 41%), fatigue (up to 40%), and vomiting (up to 28%) [36]. In a meta-analysis of Phase II and III randomized clinical trials, older patients treated with palbociclib were more likely to experience neutropenia, leukopenia, anemia, back pain, asthenia, and infections, whereas older patients treated with abemaciclib were more likely to experience neutropenia, leukopenia, anemia, and diarrhea [37]. Older patients have also been shown to have more dose reductions and delays than younger patients [38].

Regarding ICIs for metastatic melanoma, PRO data collected as part of clinical trials generally indicate that quality of life tends to be worse among patients treated with anti-CTLA4 than controls, but quality of life tends to be similar or better in those treated with anti-PD-1 compared to controls. We are not aware of published subgroup analyses in ICI trials to identify patients at risk for worse quality of life and patient-reported toxicities [39,40,41]. However, immune-related adverse events (irAEs) of ICIs are well-described, and subgroup analyses point to predisposing factors such as age, menopausal status, and pre-existing conditions. Any grade irAEs occur in about 72% of patients treated with single-agent ipilimumab, with 24% experiencing grade 3 or above [42]. Across ICIs, common irAEs include rash, pruritis, fatigue, nausea, and diarrhea. Additional irAEs include colitis, endocrinopathies, hepatitis, hypophysitis, pneumonitis, and thyroiditis [43,44]. Subgroup analyses indicate that older age was not associated with overall rate of (irAEs), but older patients were less likely to develop severe toxicities and less likely to need hospitalization, controlling for gender, performance status, season, and combination immunotherapy [45]. Sex was not associated with the rate of development of any irAEs, severe irAEs, or hospitalization in univariate or multivariate analyses [45]. Other studies have shown that menopausal status may moderate sex differences in irAEs, with premenopausal women more likely to experience toxicities [46]. Clinical predisposing factors for more severe ICI toxicity include sarcopenia, autoimmune disease, and poor kidney function [47,48,49,50], although data are mixed [45]. Because analyses have been conducted in clinical trials, data are largely lacking regarding comorbidities, autoimmune disease, and social determinants of health (e.g., insurance status). Although effectiveness and tolerability of ICIs are encouraging, there remains a need to understand how these outcomes translate to the experiences of those living with metastatic cancer.

Psychological predisposing factors also have a role in metavivorship. Depression may be a risk factor for cancer, especially lung and breast cancers [51,52,53,54,55]. Biobehavioral mechanisms for the link between depression and cancer risk have been hypothesized, including health behaviors such as tobacco use and exercise, and the impact of chronic stress on cortisol, hypothalamic-pituitary-adrenal (HPA) axis dysregulation, inflammation, and the gut microbiome [53,54,55,56]. Similar mechanisms may be involved with depression, stress, and cancer progression [57]. Depression may also impact survival. Metavivors with decreased depression during a supportive-expressive therapy intervention had a median survival twice that of those who had increased depression (medial survival 53.6 months compared to 25.1 months) [58]. In addition to mood, personality appears to be an important predisposing factor in metavivorship. Higher conscientiousness and lower neuroticism are associated with better health behaviors of exercise and diet among cancer samples [59], and adherence to cancer screenings among people without cancer [60]. Lower neuroticism is also associated with lower depression at the time of diagnosis with non-metastatic cancer, and predictive of better quality of life and lower anxiety after 6 months [61]. In a sample that included metavivors, higher neuroticism and lower extraversion, agreeableness, and conscientiousness were associated with less exercise and higher comorbidities, depression, anxiety, and symptom-related distress [62]. Spirituality has also been linked to positive coping and better quality of life among people with cancer [63], and spirituality prior to a cancer diagnosis may impact psychological outcomes after diagnosis. Among women with non-metastatic breast cancer, lower spirituality prior to diagnosis may lead to spiritual struggle and difficulty with psychological adjustment in the year after diagnosis [64]. Mood, personality, and spirituality prior to a diagnosis of metastatic cancer may be important psychological predisposing factors, yet more research is needed to determine how these factors contribute to symptoms and quality of life throughout the phases of metavivorship.

Social predisposing factors in metavivorship include social well-being, stressful life events, and socioeconomic status. Social quality of life appears to have biological mechanisms as higher social well-being was related to less leukocyte proinflammatory and pro-metastatic gene expression among non-metastatic breast cancer patients after surgery [65]. In women with metastatic or recurrent cancer, those who had no trauma history had disease-free intervals twice as long as those with a history of traumatic life events (median of 62 months compared to 31 months) [66]. Traumatic and stressful life events also predispose metavivors to develop cancer-related distress and posttraumatic stress disorder (PTSD) or subclinical posttraumatic stress symptoms (PTSS) [67]. Further research is needed to determine the impact of prior trauma on cancer-related distress among metavivors treated with targeted and ICI therapies. In a survey of cancer patients receiving chemotherapy (68% with metastatic disease), stressful life events were associated with greater cancer-related distress along with additional risk factors such as low income, lower functional status, and greater comorbidity [68]. Financial toxicity, or the financial burden of cancer and treatment on patients and their family members, also has social predisposing factors. Identified risk factors in various cancer samples include younger age, female gender, racial and ethnic minority status, unemployment, absence of health insurance, and greater out of pocket health care costs [69,70,71,72,73]. Prevention efforts may target these social predisposing factors for disease progression, survival, distress, and financial quality of life.

### 2.2. Precipitating Factors

Precipitating factors are caused by disease and treatment. These may include inter-related biological, psychological, and behavioral factors. Notably, there is a large body of research in humans and animals indicating that biological changes due to cancer and its treatment (e.g., inflammation) can cause short- and long-term psychological and behavioral changes. Inflammation is a well-established driver of metastatic disease in breast cancer and melanoma [74]. For example, neutrophils produce a wide variety of proteins that stimulate tumorigenesis as well as tumor cell proliferation, migration, and invasion [75]. Among women with metastatic breast cancer, circulating tumor cells have been found to be associated with circulating C-reactive protein (CRP) and circulating interleukin-6 (IL-6), among other inflammatory markers [76]. Melanoma is characterized by high immunogenicity and chronic inflammation in the tumor microenvironment [77]. Higher circulating levels of CRP and IL-6 prior to treatment with ICIs appear to be poor prognostic indicators among patients with metastatic melanoma [74,78,79], although evidence is mixed [80]. These findings are consistent with a large body of research demonstrating high levels of inflammation and associated behavioral changes (e.g., depression, fatigue) prior to surgery and systemic therapy for a variety of cancer types [81]. Interestingly, chronic inflammation appears to cause fatigue at least in part due to reduced cellular energy availability [82,83]. Although inflammation is among the best-studied mechanisms of psychological and behavioral changes in cancer patients, a variety of other mechanisms are also relevant. Additional research across several tumor types in human and animal studies suggests that HPA axis dysregulation as well as metabolic competition between the tumor and the rest of the organism can cause depression and fatigue [84,85,86]. These relationships appear to occur at least partially independently of inflammation [82,85]. Regarding targeted therapy for metastatic breast cancer, limited data suggest that CDK4/6 inhibitors may cause upregulation of IL-6 and IL-8 by tumor cells [87,88], although increases in systemic inflammation have not been documented to our knowledge. While all-grade fatigue is among the most common symptomatic AEs of CDK4/6 inhibitors [89,90,91,92], the mechanisms are not clear. In contrast, there is a large body of evidence that PD-1 and CTLA-4 inhibitors cause systemic inflammation in metastatic melanoma [93], including higher circulating levels of IL-1ra, IL-6, and IL-17, which are associated with irAEs such as colitis and dermatitis [94,95,96,97,98]. Further research is needed to determine whether circulating inflammatory markers are also associated with behavioral changes, such as depression and fatigue, in patients treated with ICIs.

Precipitating factors may also include psychological reactions to diagnosis and treatment of metastatic cancer. Potentially distressing events include being informed of a terminal diagnosis, sudden physical changes due to treatment, and medical emergencies due to cancer or treatment toxicities [99]. For example, irAEs can be acute medical emergencies requiring hospitalization [100]. Additional potentially distressing events include waiting for results of disease monitoring imaging studies, receiving results indicating progressive disease, and symptom interference in daily life [101]. Uncertainty regarding prognosis is also relevant given the potential for exceptional response to targeted therapies and ICIs [102]. Qualitative findings indicate that women with metastatic breast cancer report substantial psychological distress due to uncertain prognosis and treatment trajectories, and functional difficulties in social roles and responsibilities [103]. In addition, a growing body of literature suggests that cancer-related experiences can be traumatic. In one study, 69% of metastatic lung cancer patients indicated that their diagnosis and cancer treatment qualified as traumatic stressors per Diagnostic and Statistical Manual of Mental Disorders-5th Edition (DSM-5) criteria. Moreover, those who reported traumatic cancer-related experiences also reported greater distress and worry about progression [104]. In a sample including 42% with metastatic disease, cancer-related traumatic stress symptoms were associated with worse sleep disturbance and pain [105]. These findings suggest that precipitating psychological reactions to potentially traumatic cancer-related events may contribute to PROs such as psychological distress and sleep disturbance. The impact of cancer-related events and emotional responses on PROs for metavivors should be examined in future research.

Social and environmental precipitating factors may vary in their impact throughout the early and chronic phases and include changes to social roles and functioning due to cancer and treatment, such as changes to work responsibilities or employment. One study of women with metastatic breast cancer found that 45% stopped working due to illness, and 58% reported a change in employment [106]; however, data were collected between 2006 and 2008, and rapidly changing treatment paradigms may have a different impact on employment now. Another study found that 87% of metavivors across diagnoses stopped working within 6 months of diagnosis [107]. Changes to employment can lead to financial burden and inadequate health insurance coverage, which can increase distress, reduce appropriate healthcare utilization, and negatively impact symptoms and quality of life [108]. Further research is needed to determine the impact of targeted therapies and ICIs on employment and subsequent financial quality of life among metavivors. Metavivors can also experience changes to engagement with important social activities, and changes in the amount of support provided by others. Disrupted social and recreational activities are associated with greater depressive symptoms among breast cancer metavivors [109]. Support provided by friends, family, and others may initially increase in the acute and early phases of metavivorship, but then decline over time [3,110]. Social support either with practical needs or emotional support is important for symptom management, and low social support is even associated with mortality [111,112,113].

### 2.3. Perpetuating Factors

Perpetuating factors are longer-term biological, psychological, behavioral, and social changes due to precipitating factors that can sustain or exacerbate poor outcomes. Perpetuating factors can start as precipitating factors and become chronic, or they can emerge in response to precipitating factors. Biological perpetuating factors can result from disease or treatment. Tumor(s) may have an ongoing biological impact in metavivors with stable or progressive disease. Interestingly, however, chronic circulating markers of inflammation (i.e., IL-6, CXCL1) were observed in mouse models even after tumor resection, suggesting long-term changes due to the cancer or surgery even when the tumor is no longer present [114]. Moreover, tumor mass was associated with anxiety-like behavioral changes, even after resection [114]. These findings suggest that metavivors with complete response may have chronic biological and behavioral changes due to cancer. Regarding perpetuating biological factors due to treatment, we are unaware of any data regarding long-term or late effects of anti-CDK4/6 or anti-PI3K agents in metastatic breast cancer. Data on long-term and late effects of ICIs in melanoma patients are limited to a few case studies demonstrating new irAEs after completion or discontinuation of therapy [115,116,117]. In light of the well-known inflammatory mechanisms of irAEs during ICIs [98], chronic inflammation following treatment is a plausible pathway. Notably, the accumulation of multiple or chronic low-grade toxicities can have a greater impact than time-limited, severe adverse events [118]. As the number of metavivors increases, long-term and late effects are an important area of study.

Psychological perpetuating factors are cognitive and behavioral changes made in response to precipitating factors. While these changes may help manage symptoms in the short-term, some can contribute to sustained symptoms and negatively impact quality of life in the acute and chronic phases of metavivorship. For example, increased attention to unusual physical sensations may be an impetus for seeking medical care and play an important role in an initial cancer diagnosis and monitoring during active treatment; however, continued hypervigilance can lead to chronic anxiety and panic [119]. The prevalence of panic attacks in metavivors is unknown. However, people with cancer are twice as likely to experience panic attacks as those in the general population [120]. PTSS such as intrusive thoughts about aversive cancer-related events, avoiding reminders of cancer, negative cognitive appraisals (e.g., fatalistic or hopeless beliefs), and hyperarousal (i.e., heightened psychophysiological reactivity) are experienced to some extent by most people who have had cancer [121], although the prevalence of cancer-related PTSD generally falls around 10% [67,101]. In metavivors, cancer-related PTSD may be more common, with small sample sizes of metastatic breast cancer and melanoma showing prevalence rates between 35 and 52% [122,123,124]. In general, cancer-related distress appears to peak at diagnosis and during active treatment, and decline over time. However, more severe distress predicts continued high symptom burden and later diagnosis of cancer-related PTSD, indicating that for some patients, symptoms do not subside [125,126]. Cognitive predictors of cancer-related distress include illness perceptions and coping. For example, among lung cancer metavivors [127] and newly diagnosed early stage breast cancer patients [128], depression and anxiety are associated with fatalistic beliefs about the controllability of cancer, beliefs that emotions contribute to the development of cancer, and anxious preoccupation (i.e., worrying). There is a growing body of evidence that these cognitive and behavioral responses are amenable to change after intervention, with subsequent improvement in PROs (e.g., distress, fatigue, sleep disturbance, cognitive impairment) [129,130,131].

There are several social and environmental factors that can perpetuate metavivors’ PROs. Social support across all stages of metavivorship has important benefits for metavivors, including increased hope and lowered depressive symptoms [132]. However, several social challenges may develop, including social constraint from loved ones who find it too painful to discuss a range of important topics from the cancer itself to death and dying [103]. Metavivors may also choose to join metavivor-focused support groups. Although many metavivors seek support groups to gain comfort and companionship, they may also hear about traumatic medical events and grieve the deaths of fellow group members [133]. Metavivors also experience changes to their socioeconomic environment, including financial toxicity. Metavivors are particularly vulnerable to financial toxicity because targeted and ICI treatments are among the most expensive cancer therapies available [134,135]. Financial toxicity, in turn, places metavivors at greater risk for psychological distress compared to those with early stage disease [136]. Moreover, financial toxicity can lead to medication non-adherence and underutilization of health care services [136], which can exacerbate symptoms, morbidity, and mortality.

## 3. Implications

Clinical identification of predisposing, precipitating, and perpetuating factors affecting metavivors’ PROs may lead to more effective prevention, management, and treatment. Comprehensive biopsychosocial screenings are a key tool for this purpose [137]. Biopsychosocial screenings can take the form of self-report questionnaires, medical tests and labs, data from wearables (e.g., fitness trackers), and/or an integration of data from multiple sources. Clinicians can use biopsychosocial screenings to identify metavivors’ predisposing factors at the point of diagnosis or earlier (e.g., during cancer screenings, during prior treatments for non-metastatic disease), as well as to identify precipitating and perpetuating factors as they change throughout metavivorship. Thus, routine biopsychosocial screenings can identify ongoing and shifting opportunities for intervention. As a stark example, a study of more than 734,000 patients diagnosed with various cancers found that being married was more strongly associated with longer survival than chemotherapy after accounting for disease and treatment characteristics, and unmarried individuals were more likely to have metastatic disease [138]. Knowing this, marital status is one predisposing social factor that can be used to identify metavivors who could benefit from early referrals to services designed to enhance social support in an effort to mitigate these effects. This is true of other known predisposing factors as well (e.g., age, socioeconomic status, history of psychopathology). Notably, existing systems are already primed to incorporate biopsychosocial screening into metavivors’ care. This is due in part to a major shift in patient-centered cancer care that occurred in 2015 with implementation of the American College of Surgeons Commission on Cancer’s standard requiring routine distress screening in all cancer programs. As a result, cancer programs across the US were required to determine when, how, and how often to solicit PRO data related to distress from individuals receiving cancer care [139]. Implementation of this standard looks different across programs depending on a host of factors including treatment setting, types of cancers treated, institutional resources, and clinic workflows. Yet as a result, cancer programs have laid the groundwork to incorporate additional measures and data sources into routine distress screening protocols to form more comprehensive biopsychosocial screenings.

To facilitate biopsychosocial screening protocols, cancer programs may consider leveraging the rapid technological advances of the past several decades, such as eHealth. eHealth is the use of information and communication technologies to facilitate care, and examples include online patient portals, telemedicine, and smartphone apps [140,141]. A recent trial underscores the potential benefits of using eHealth to routinely monitor patients’ side effects during cancer treatments; Basch and colleagues [142] randomized over 700 patients with advanced cancer undergoing chemotherapy to report on treatment side effects either on a weekly basis using tablet computers or at intervals determined by their providers. Nurses were alerted to severe or worsening symptoms by email. Relative to usual care, routine eHealth PRO monitoring was associated with less decline in quality of life, fewer emergency room admissions, longer treatment with chemotherapy, and longer survival (median five months survival benefit for metavivors) [142,143]. The observed survival benefit was later replicated in advanced lung cancer [144]. The benefits of routine eHealth PRO monitoring are attributed in part to early detection of side effects during treatment. In some cases, this resulted in early detection and resolution of potentially dangerous complications, resulting in better tolerability of chemotherapy and, therefore, a higher dose of treatment. As applied to the 3P Model, early detection of precipitating factors (i.e., side effects) using routine eHealth PRO monitoring led to early intervention, which may have buffered against the development of perpetuating factors and thus improved outcomes. Since this seminal study, multiple eHealth systems have been developed for monitoring PROs and other patient-generated data (e.g., from wearables) [140,141,145], and several cancer centers have integrated routine eHealth PRO monitoring into patient portals and electronic medical records to promote patient–provider communication, shared-decision making, and patient-centered care [146,147,148]. Importantly, eHealth offers the flexibility to tailor biopsychosocial screenings to individual patient and treatment-related characteristics (e.g., predisposing factors, precipitating factors such as disease characteristics and known side effects of treatment regimens), further promoting patient-centered care [137].

Research to identify treatments for reducing precipitating and perpetuating factors among metavivors is relatively limited [149]. Existing studies have sought to improve patient education and communication with medical providers at the point of metastatic diagnosis and during treatment using decision aids [150,151,152,153,154,155,156,157], communication aids [153,154], question prompt lists [150,151,152], and educational videos and handouts [155,157]. Results are mostly positive, indicating the efficacy of these interventions for improving patient-centered outcomes such as communication. However, several studies were conducted prior to recent treatment advances and improvement of metavivors’ clinical outcomes. Thus, more research is needed to determine how education and communication interventions may best support metavivors in the context of targeted and ICI treatments and long-term metavivorship. Medical interventions are used in supportive and palliative care to manage symptoms such as depression, anxiety, pain, and sleep disturbance. Integration of early palliative care with tumor-directed treatment has been encouraged for a person-centered approach [158]. Compared to palliative care consultations, systematic integration of palliative care in oncology has shown a greater impact on quality of life for advanced cancer patients [159]. Current evidence-based interdisciplinary treatments for palliative care of cancer-related symptoms are presented in clinical practice guidelines such as from the American Society of Clinical Oncology [160] and National Comprehensive Cancer Network [161]. Ongoing research efforts are also exploring new opportunities for medical management of cancer-related distress and symptom burden. For example, emerging evidence suggests that a single high dose of psilocybin may produce lasting improvement in depression and anxiety in patients with terminal cancer [162], and cannabinoids are under investigation to reduce overall symptom burden among patients with advanced cancer [163]. Other studies mostly grounded in cognitive-behavioral therapy have sought to directly improve PROs including distress [164,165,166,167,168,169], quality of life [170,171,172,173,174,175], and side effects of metastatic cancer and its treatments [176,177,178,179,180]. Results suggest that evidence-based interventions are efficacious for improving PROs among metavivors. However, as before, more work is needed to verify that interventions to directly improve metavivors’ PROs are efficacious in the context of cutting-edge treatments and long-term metavivorship.

Although some metavivors are living longer with their disease, metastatic cancer remains a terminal diagnosis; thus, advance care planning (ACP) including clarifying treatment goals and formal advance directive documentation may be helpful for metavivors and their family caregivers. ACP remains underutilized along with supportive and palliative care services [181]. Providers may hesitate to initiate ACP discussions due to concern about causing distress; however, a large prospective study of patients with advanced cancer and family caregivers found that end-of-life discussions with medical providers were not associated with increased risk of depression or worry [182]. In fact, discussions between patients and providers regarding goals of care were associated with fewer hospital admissions and less aggressive treatment near end-of-life [183,184], which may improve quality of life and reduce financial toxicity [185]. Bereaved family members also have better psychological adjustment when patients undergo less aggressive treatments near end-of-life [182]. The majority of research on ACP involves hospitalized patients and those near end-of-life [183,184,185,186]; however, goals of care can be an ongoing and evolving discussion throughout metavivorship. Further research is needed to determine the optimal timing of ACP discussions for metavivors and their family caregivers. In addition, a systematic review found disparities in access to hospice care and inconsistencies between end-of-life care and patient preferences among Hispanic and African American patients with advanced cancer [187]. These findings highlight a need for future research to improve equity in ACP discussions and end-of-life care. Certain cultural or religious influences can also impact ACP discussions and must be considered to ensure a holistic approach to end-of-life care [188]. An extensive discussion of ACP perceptions, barriers, and best practices is available from the National Cancer Institute [189].

As a final note, it is important to recognize that that are several psychological and behavioral protective factors that can improve PROs in metavivorship. Despite the challenges of living with a terminal illness, metavivors demonstrate incredible resilience in the face of uncertainty about the future. Practicing mindfulness, living consistently with one’s values, physical exercise, and a nutritious diet all show promise for improving PROs in cancer [190,191]. Metavivors may also cultivate meaning through their choice of attitude, connections with others and the world, participation in activities, and considerations of their legacy [192,193]. Metavivors may engage in positive reinterpretation such as seeing the “silver lining” of difficult situations, emphasizing things that are within their control, and expressing gratitude for the everyday [194]. Hope (i.e., the desire or expectation for a positive outcome or lack of a negative outcome) is another important positive psychological construct that may help to explain how individuals adapt in the context of terminal illness. Literature suggests that hope persists in metavivors regardless of proximity to death [195,196,197], and hope is dynamic such that people who report more hope are more likely to incorporate relevant contextual information into goal appraisal, adjust their goals or develop new attainable goals, and work toward these goals [198]. Positive reinterpretation and optimism do have limits, especially when employed to avoid aversive yet reality-based thoughts and emotions such as fear and grief. Mindfulness and self-compassion may attenuate the impact of negative events while validating emotional experiences [199,200,201,202]. Among patients with hematologic cancer recovering from hematopoietic stem cell transplantation (HSCT), mindfulness and acceptance of thoughts and emotions was positively associated with psychological outcomes following HSCT [203]. Among patients with advanced cancer, there is growing evidence for the efficacy of meaning-enhancing interventions (e.g., meaning-centered psychotherapy, supportive expressive therapy) for improving PROs such as spiritual well-being, sense of meaning, and quality of life [149,204,205]. Thus, interventions focused on cultivating these protective factors may be a critical complement to interventions designed to reduce precipitating and perpetuating factors in metavivorship care.

## 4. Conclusions

In conclusion, metavivorship presents several challenges that differ from patients and survivors with earlier stage disease. Metavivors treated with novel targeted and ICI therapies are living longer with their disease yet are faced with uncertainty in their prognosis and length of survival, acute and chronic symptoms, psychological distress, and financial toxicity. Clinical trials and retrospective medial record reviews provide information regarding clinician-rated adverse events. However, further research is needed regarding patient-reported outcomes and factors that impact important outcomes for metavivors. We provide a biopsychosocial conceptualization of predisposing, precipitating, and perpetuating factors for the challenges of metavivorship. Prevention efforts may target those with risk factors that predispose metavivors to develop progressive disease, reduced survival, symptoms, and financial toxicity. These outcomes may be mitigated by managing precipitating events such as immune-related adverse events, acute psychological distress, and social challenges. After prevention and management efforts, treatments can be implemented for perpetuating responses such as cumulative effects of treatment, changes to cognitive and behavioral patterns, and social constraints and financial burden. Specific recommendations for future research and clinical interventions include biopsychosocial screening, monitoring of patient-reported outcomes, education and communication interventions, interdisciplinary symptom management, advance care planning, and behavioral interventions to cultivate psychological resilience. Such efforts have the potential to have a high impact on quality of life for the growing population of metavivors.

## References

[1] Hewitt M., Greenfield S., Stovall E. (2006). From Cancer Patient to Cancer Survivor: Lost in Transition.

[2] Davis K. Opportunities and barriers to high-quality care in metastatic breast cancer from a patient perspective. Proceedings of the ASCO 2019.

[3] Spoozak L., Wulff-Burchfield E., Brooks J.V. (2020). Rallying cry from the place in between. JCO Oncol. Pract..

[4] Jacobsen P.B., Nipp R.D., Ganz P.A. (2017). Addressing the survivorship care needs of patients receiving extended cancer treatment. Am. Soc. Clin. Oncol. Educ. Book.

[5] Sundquist M., Brudin L., Tejler G. (2017). Improved survival in metastatic breast cancer 1985–2016. Breast.

[6] Berk-Krauss J., Stein J.A., Weber J., Polsky D., Geller A.C. (2020). New systematic therapies and trends in cutaneous melanoma deaths among US whites, 1986–2016. Am. J. Public Health.

[7] Roskoski R. (2020). Properties of FDA-approved small molecule protein kinase inhibitors: A 2020 update. Pharmacol. Res..

[8] Darvin P., Toor S.M., Sasidharan Nair V., Elkord E. (2018). Immune checkpoint inhibitors: Recent progress and potential biomarkers. Exp. Mol. Med..

[9] US. Department of Health and Human Services Food and Drug Administration (2009). Patient-reported outcome measures: Use in medical product development to support labeling claims. Guidance for Industry.

[10] Spielman A.J., Caruso L.S., Glovinsky P.B. (1987). A behavioral perspective on insomnia treatment. Psychiatr. Clin. N. Am..

[11] Wright C.D., Tiani A.G., Billingsley A.L., Steinman S.A., Larkin K.T., McNeil D.W. (2019). A framework for understanding the role of psychological processes in disease development, maintenance, and treatment: The 3P-disease model. Front. Psychol..

[12] Wacholder S., Hartge P., Prentice R., Garcia-Closas M., Feigelson H.S., Diver W.R., Thun M.J., Cox D.G., Hankinson S.E., Kraft P. (2010). Performance of common genetic variants in breast-cancer risk models. N. Engl. J. Med..

[13] Simapivapan P., Boltong A., Hodge A. (2016). To what extent is alcohol consumption associated with breast cancer recurrence and second primary breast cancer?: A systematic review. Cancer Treat. Rev..

[14] Barone I., Giordano C., Bonofiglio D., Ando S., Catalano S. (2020). The weight of obesity in breast cancer progression and metastasis: Clinical and molecular perspectives. Semin. Cancer Biol..

[15] Hanson H.A., Smith K.R., Zimmer Z. (2015). Reproductive history and later-life comorbidity trajectories: A medicare-lnked cohort study from the Utah population database. Demography.

[16] Carr S., Smith C., Wernberg J. (2020). Epidemiology and risk factors of melanoma. Surg. Clin. N. Am..

[17] Jing L., Su L., Ring B.Z. (2014). Ethnic background and genetic variation in the evaluation of cancer risk: A systematic review. PLoS ONE.

[18] Franzoi M.A., Eiger D., Ameye L., Ponde N., Caparica R., De Angelis C., Brandao M., Desmedt C., Di Cosimo S., Kotecki N. (2020). Clinical implications of body mass index in metastatic breast cancer patients treated with abemaciclib and endocrine therapy. J. Natl. Cancer Inst..

[19] Sattar J., Kartolo A., Hopman W.M., Lakoff J.M., Baetz T. (2019). The efficacy and toxicity of immune checkpoint inhibitors in a real-world older patient population. J. Geriatr. Oncol..

[20] Conforti F., Pala L., Bagnardi V., De Pas T., Martinetti M., Viale G., Gelber R.D., Goldhirsch A. (2018). Cancer immunotherapy efficacy and patients’ sex: A systematic review and meta-analysis. Lancet Oncol..

[21] Crocetti E., Fancelli L., Manneschi G., Caldarella A., Pimpinelli N., Chiarugi A., Nardini P., Buzzoni C. (2016). Melanoma survival: Sex does matter, but we do not know how. Eur. J. Cancer Prev..

[22] McQuade J.L., Daniel C.R., Hess K.R., Mak C., Wang D.Y., Rai R.R., Park J.J., Haydu L.E., Spencer C., Wongchenko M. (2018). Association of body-mass index and outcomes in patients with metastatic melanoma treated with targeted therapy, immunotherapy, or chemotherapy: A retrospective, multicohort analysis. Lancet Oncol..

[23] Gingrich A.A., Sauder C.A.M., Goldfarb M., Li Q., Wun T., Keegan T.H.M. (2020). Disparities in the occurrence of late effects following treatment among adolescent and young adult melanoma survivors. Cancer Epidemiol. Biomark. Prev..

[24] Chu M.P., Li Y., Ghosh S., Sass S., Smylie M., Walker J., Sawyer M.B. (2020). Body composition is prognostic and predictive of ipilimumab activity in metastatic melanoma. J. Cachexia Sarcopenia Muscle.

[25] Naik G.S., Waikar S.S., Johnson A.E.W., Buchbinder E.I., Haq R., Hodi F.S., Schoenfeld J.D., Ott P.A. (2019). Complex inter-relationship of body mass index, gender and serum creatinine on survival: Exploring the obesity paradox in melanoma patients treated with checkpoint inhibition. J. Immunother. Cancer.

[26] Haque W., Verma V., Butler E.B., Teh B.S. (2019). Racial and socioeconomic disparities in the delivery of immunotherapy for metastatic melanoma in the United States. J. Immunother..

[27] Li D., Duan H., Jiang P., Jiang X., He Z., Guo C., Mou Y. (2020). Trend and socioeconomic disparities in survival outcome of metastatic melanoma after approval of immune checkpoint inhibitors: A population-based study. Am. J. Transl. Res..

[28] Basch E., Jia X., Heller G., Barz A., Sit L., Fruscione M., Appawu M., Iasonos A., Atkinson T., Goldfarb S. (2009). Adverse symptom event reporting by patients vs clinicians: Relationships with clinical outcomes. J. Natl. Cancer Inst..

[29] Petersen M.A., Larsen H., Pedersen L., Sonne N., Groenvold M. (2006). Assessing health-related quality of life in palliative care: Comparing patient and physician assessments. Eur. J. Cancer.

[30] Guan M., Gresham G., Shinde A., Lapite I., Gong J., Placencio-Hickok V.R., Forrest C.B., Hendifar A.E. (2020). Priority rankings of patient-reported outcomes for pancreatic ductal adenocarcinoma: A comparison of patient and physician perspectives. J. Natl. Compr. Cancer Netw..

[31] Pakhomov S.V., Jacobsen S.J., Chute C.G., Roger V.L. (2008). Agreement between patient-reported symptoms and their documentation in the medical record. Am. J. Manag. Care.

[32] Finn R.S., Crown J.P., Ettl J., Schmidt M., Bondarenko I.M., Lang I., Pinter T., Boer K., Patel R., Randolph S. (2016). Efficacy and safety of palbociclib in combination with letrozole as first-line treatment of ER-positive, HER2-negative, advanced breast cancer: Expanded analyses of subgroups from the randomized pivotal trial PALOMA-1/TRIO-18. Breast Cancer Res..

[33] Kish J.K., Ward M.A., Garofalo D., Ahmed H.V., McRoy L., Laney J., Zanotti G., Braverman J., Yu H., Feinberg B.A. (2018). Real-world evidence analysis of palbociclib prescribing patterns for patients with advanced/metastatic breast cancer treated in community oncology practice in the USA one year post approval. Breast Cancer Res..

[34] Lee K.W.C., Lord S., Finn R.S., Lim E., Martin A., Loi S., Lynch J., Friedlander M., Lee C.K. (2019). The impact of ethnicity on efficacy and toxicity of cyclin D kinase 4/6 inhibitors in advanced breast cancer: A meta-analysis. Breast Cancer Res. Treat..

[35] Rugo H.S., Turner N.C., Finn R.S., Joy A.A., Verma S., Harbeck N., Masuda N., Im S.A., Huang X., Kim S. (2018). Palbociclib plus endocrine therapy in older women with HR+/HER2- advanced breast cancer: A pooled analysis of randomised PALOMA clinical studies. Eur. J. Cancer.

[36] Ponde N., Wildiers H., Awada A., de Azambuja E., Deliens C., Lago L.D. (2020). Targeted therapy for breast cancer in older patients. J. Geriatr. Oncol..

[37] Omarini C., Piacentini F., Sperduti I., Barbolini M., Isca C., Toss A., Cortesi L., Barbieri E., Dominici M., Moscetti L. (2020). Combined endocrine approaches vs endocrine therapy alone as first line treatment in elderly patients with hormone receptor-positive, HER2 negative, advanced breast cancer: To prescribe for the patient or the physician? A meta-analysis of phase II and III randomized clinical trials. BMC Cancer.

[38] Clifton K., Min Y., Kimmel J., Litton J., Tripathy D., Karuturi M. (2019). Progression-free survival (PFS) and toxicities of palbociclib in a geriatric population. Breast Cancer Res. Treat..

[39] Coens C., Suciu S., Chiarion-Sileni V., Grob J.J., Dummer R., Wolchok J.D., Schmidt H., Hamid O., Robert C., Ascierto P.A. (2017). Health-related quality of life with adjuvant ipilimumab versus placebo after complete resection of high-risk stage III melanoma (EORTC 18071): Secondary outcomes of a multinational, randomised, double-blind, phase 3 trial. Lancet Oncol..

[40] Long G.V., Atkinson V., Ascierto P.A., Robert C., Hassel J.C., Rutkowski P., Savage K.J., Taylor F., Coon C., Gilloteau I. (2016). Effect of nivolumab on health-related quality of life in patients with treatment-naive advanced melanoma: Results from the phase III CheckMate 066 study. Ann. Oncol..

[41] Petrella T.M., Robert C., Richtig E., Miller W.H., Masucci G.V., Walpole E., Lebbe C., Steven N., Middleton M.R., Hille D. (2017). Patient-reported outcomes in KEYNOTE-006, a randomised study of pembrolizumab versus ipilimumab in patients with advanced melanoma. Eur. J. Cancer.

[42] Bertrand A., Kostine M., Barnetche T., Truchetet M.E., Schaeverbeke T. (2015). Immune related adverse events associated with anti-CTLA-4 antibodies: Systematic review and meta-analysis. BMC Med..

[43] Weber J.S., Yang J.C., Atkins M.B., Disis M.L. (2015). Toxicities of immunotherapy for the practitioner. J. Clin. Oncol..

[44] Michot J.M., Bigenwald C., Champiat S., Collins M., Carbonnel F., Postel-Vinay S., Berdelou A., Varga A., Bahleda R., Hollebecque A. (2016). Immune-related adverse events with immune checkpoint blockade: A comprehensive review. Eur. J. Cancer.

[45] Shah K.P., Song H., Ye F., Moslehi J.J., Balko J.M., Salem J.E., Johnson D.B. (2020). Demographic factors associated with toxicity in patients treated with anti-programmed cell death-1 therapy. Cancer Immunol. Res..

[46] Duma N., Abdel-Ghani A., Yadav S., Hoversten K.P., Reed C.T., Sitek A.N., Enninga E.A.L., Paludo J., Aguilera J.V., Leventakos K. (2019). Sex differences in tolerability to anti-programmed cell death protein 1 therapy in patients with metastatic melanoma and non-small cell lung cancer: Are we all equal?. Oncologist.

[47] Daly L.E., Power D.G., O’Reilly A., Donnellan P., Cushen S.J., O’Sullivan K., Twomey M., Woodlock D.P., Redmond H.P., Ryan A.M. (2017). The impact of body composition parameters on ipilimumab toxicity and survival in patients with metastatic melanoma. Br. J. Cancer..

[48] Kartolo A., Sattar J., Sahai V., Baetz T., Lakoff J.M. (2018). Predictors of immunotherapy-induced immune-related adverse events. Curr. Oncol..

[49] Kahler K.C., Eigentler T.K., Gesierich A., Heinzerling L., Loquai C., Meier F., Meiss F., Pfohler C., Schlaak M., Terheyden P. (2018). Ipilimumab in metastatic melanoma patients with pre-existing autoimmune disorders. Cancer Immunol. Immunother..

[50] Menzies A.M., Johnson D.B., Ramanujam S., Atkinson V.G., Wong A.N.M., Park J.J., McQuade J.L., Shoushtari A.N., Tsai K.K., Eroglu Z. (2017). Anti-PD-1 therapy in patients with advanced melanoma and preexisting autoimmune disorders or major toxicity with ipilimumab. Ann. Oncol..

[51] Chen Y.H., Lin H.C. (2011). Increased risk of cancer subsequent to severe depression—A nationwide population-based study. J. Affect. Disord..

[52] Goldacre M.J., Wotton C.J., Yeates D., Seagroatt V., Flint J. (2007). Cancer in people with depression or anxiety: Record-linkage study. Soc. Psychiatry Psychiatr. Epidemiol..

[53] Wang Y.H., Li J.Q., Shi J.F., Que J.Y., Liu J.J., Lappin J.M., Leung J., Ravindran A.V., Chen W.Q., Qiao Y.L. (2019). Depression and anxiety in relation to cancer incidence and mortality: A systematic review and meta-analysis of cohort studies. Mol. Psychiatry.

[54] Jia Y., Li F., Liu Y.F., Zhao J.P., Leng M.M., Chen L. (2017). Depression and cancer risk: A systematic review and meta-analysis. Public Health.

[55] Gross A.L., Gallo J.J., Eaton W.W. (2010). Depression and cancer risk: 24 years of follow-up of the Baltimore Epidemiologic Catchment Area sample. Cancer Causes Control.

[56] Bortolato B., Hyphantis T.N., Valpione S., Perini G., Maes M., Morris G., Kubera M., Köhler C.A., Fernandes B.S., Stubbs B. (2017). Depression in cancer: The many biobehavioral pathways driving tumor progression. Cancer Treat. Rev..

[57] Shin K.J., Lee Y.J., Yang Y.R., Park S., Suh P.G., Follo M.Y., Cocco L., Ryu S.H. (2016). Molecular mechanisms underlying psychological stress and cancer. Curr. Pharm. Des..

[58] Giese-Davis J., Collie K., Rancourt K.M., Neri E., Kraemer H.C., Spiegel D. (2011). Decrease in depression symptoms is associated with longer survival in patients with metastatic breast cancer: A secondary analysis. J. Clin. Oncol..

[59] Rochefort C., Hoerger M., Turiano N.A., Duberstein P. (2019). Big Five personality and health in adults with and without cancer. J. Health Psychol..

[60] Aschwanden D., Gerend M.A., Luchetti M., Stephan Y., Sutin A.R., Terracciano A. (2019). Personality traits and preventive cancer screenings in the Health Retirement Study. Prev. Med..

[61] Hulbert-Williams N., Neal R., Morrison V., Hood K., Wilkinson C. (2012). Anxiety, depression and quality of life after cancer diagnosis: What psychosocial variables best predict how patients adjust?. Psycho-Oncol..

[62] Morgan S., Cooper B., Paul S., Hammer M.J., Conley Y.P., Levine J.D., Miaskowski C., Dunn L.B. (2017). Association of personality profiles with depressive, anxiety, and cancer-related symptoms in patients undergoing chemotherapy. Personal. Individ. Differ..

[63] Puchalski C.M. (2012). Spirituality in the cancer trajectory. Ann. Oncol..

[64] Gall T.L., Kristjansson E., Charbonneau C., Florack P. (2009). A longitudinal study on the role of spirituality in response to the diagnosis and treatment of breast cancer. J. Behav. Med..

[65] Jutagir D.R., Blomberg B.B., Carver C.S., Lechner S.C., Timpano K.R., Bouchard L.C., Gudenkauf L.M., Jacobs J.M., Diaz A., Lutgendorf S.K. (2017). Social well-being is associated with less pro-inflammatory and pro-metastatic leukocyte gene expression in women after surgery for breast cancer. Breast Cancer Res. Treat..

[66] Palesh O., Butler L.D., Koopman C., Giese-Davis J., Carlson R., Spiegel D. (2007). Stress history and breast cancer recurrence. J. Psychosom. Res..

[67] Cordova M.J., Riba M.B., Spiegel D. (2017). Post-traumatic stress disorder and cancer. Lancet Psychiatry.

[68] Langford D.J., Cooper B., Paul S., Humphreys J., Keagy C., Conley Y.P., Hammer M.J., Levine J.D., Wright F., Melisko M. (2017). Evaluation of coping as a mediator of the relationship between stressful life events and cancer-related distress. Health Psychol..

[69] Lentz R., Benson A.B., Kircher S. (2019). Financial toxicity in cancer care: Prevalence, causes, consequences, and reduction strategies. J. Surg. Oncol..

[70] Carrera P.M., Kantarjian H.M., Blinder V.S. (2018). The financial burden and distress of patients with cancer: Understanding and stepping-up action on the financial toxicity of cancer treatment. CA Cancer J. Clin..

[71] Gordon L.G., Merollini K.M.D., Lowe A., Chan R.J. (2017). A systematic review of financial toxicity among cancer survivors: We can’t pay the co-pay. Patient.

[72] Hastert T.A., Banegas M.P., Hamel L.M., Reed A.R., Baird T., Beebe-Dimmer J.L., Schwartz A.G. (2019). Race, financial hardship, and limiting care due to cost in a diverse cohort of cancer survivors. J. Cancer Surviv..

[73] de Souza J.A., Yap B.J., Wroblewski K., Blinder V., Araújo F.S., Hlubocky F.J., Nicholas L.H., O’Connor J.M., Brockstein B., Ratain M.J. (2017). Measuring financial toxicity as a clinically relevant patient-reported outcome: The validation of the COmprehensive Score for financial Toxicity (COST). Cancer.

[74] Laino A.S., Woods D., Vassallo M., Qian X., Tang H., Wind-Rotolo M., Weber J. (2020). Serum interleukin-6 and C-reactive protein are associated with survival in melanoma patients receiving immune checkpoint inhibition. J. Immunother. Cancer.

[75] Liang W., Ferrara N. (2016). The complex role of neutrophils in tumor angiogenesis and metastasis. Cancer Immunol. Res..

[76] Lohmann A.E., Dowling R.J.O., Ennis M., Amir E., Elser C., Brezden-Masley C., Vandenberg T., Lee E., Fazaee K., Stambolic V. (2018). Association of metabolic, inflammatory, and tumor markers with circulating tumor cells in metastatic breast cancer. JNCI Cancer Spectr..

[77] Umansky V., Blattner C., Gebhardt C., Utikal J. (2017). CCR5 in recruitment and activation of myeloid-derived suppressor cells in melanoma. Cancer Immunol. Immunother..

[78] Nakamura Y., Kitano S., Takahashi A., Tsutsumida A., Namikawa K., Tanese K., Abe T., Funakoshi T., Yamamoto N., Amagai M. (2016). Nivolumab for advanced melanoma: Pretreatment prognostic factors and early outcome markers during therapy. Oncotarget.

[79] Bjoern J., Juul Nitschke N., Zeeberg Iversen T., Schmidt H., Fode K., Svane I.M. (2016). Immunological correlates of treatment and response in stage IV malignant melanoma patients treated with Ipilimumab. Oncoimmunology.

[80] Yamazaki N., Kiyohara Y., Uhara H., Iizuka H., Uehara J., Otsuka F., Fujisawa Y., Takenouchi T., Isei T., Iwatsuki K. (2017). Cytokine biomarkers to predict antitumor responses to nivolumab suggested in a phase 2 study for advanced melanoma. Cancer Sci..

[81] Lutgendorf S.K., Weinrib A.Z., Penedo F., Russell D., DeGeest K., Costanzo E.S., Henderson P.J., Sephton S.E., Rohleder N., Lucci J.A. (2008). Interleukin-6, cortisol, and depressive symptoms in ovarian cancer patients. J. Clin. Oncol..

[82] Lacourt T.E., Vichaya E.G., Chiu G.S., Dantzer R., Heijnen C.J. (2018). The high costs of low-grade inflammation: Persistent fatigue as a consequence of reduced cellular-energy availability and non-adaptive energy expenditure. Front. Behav. Neurosci..

[83] Lacourt T.E., Vichaya E.G., Escalante C., Manzullo E.F., Gunn B., Hess K.R., Heijnen C.J., Dantzer R. (2018). An effort expenditure perspective on cancer-related fatigue. Psychoneuroendocrinology.

[84] Grossberg A.J., Vichaya E.G., Gross P.S., Ford B.G., Scott K.A., Estrada D., Vermeer D.W., Vermeer P., Dantzer R. (2020). Interleukin 6-independent metabolic reprogramming as a driver of cancer-related fatigue. Brain Behav. Immun..

[85] Grossberg A.J., Vichaya E.G., Christian D.L., Molkentine J.M., Vermeer D.W., Gross P.S., Vermeer P.D., Lee J.H., Dantzer R. (2018). Tumor-associated fatigue in cancer patients develops independently of IL1 signaling. Cancer Res..

[86] Weinrib A.Z., Sephton S.E., Degeest K., Penedo F., Bender D., Zimmerman B., Kirschbaum C., Sood A.K., Lubaroff D.M., Lutgendorf S.K. (2010). Diurnal cortisol dysregulation, functional disability, and depression in women with ovarian cancer. Cancer.

[87] Kettner N.M., Bui T., Chen X., Hunt K.K., Tripathy D., Keyomarsi K. Role of IL-6 in promoting endocrine therapy and palbociclib resistance estrogen receptor positive breast cancer cells. Proceedings of the Cancer Research Symposium.

[88] Martinez Bueno A., Bertran Aramillo J., Garcia Mosquera J.J., Gimenez Capitan A., Codony-Servat J., Aguilar A., Garcia Roman S., Mayo de las Casas C., Viteri S., Molina M.A. (2019). Palbociclib-induced senescence upregulates the expression of IL-8 and may enhance the response to immunotherapy. Ann. Oncol..

[89] Finn R.S., Martin M., Rugo H.S., Jones S., Im S.A., Gelmon K., Harbeck N., Lipatov O.N., Walshe J.M., Moulder S. (2016). Palbociclib and letrozole in advanced breast cancer. N. Engl. J. Med..

[90] Cristofanilli M., Turner N.C., Bondarenko I., Ro J., Im S.A., Masuda N., Colleoni M., DeMichele A., Loi S., Verma S. (2016). Fulvestrant plus palbociclib versus fulvestrant plus placebo for treatment of hormone-receptor-positive, HER2-negative metastatic breast cancer that progressed on previous endocrine therapy (PALOMA-3): Final analysis of the multicentre, double-blind, phase 3 randomised controlled trial. Lancet Oncol..

[91] Goetz M.P., Toi M., Campone M., Sohn J., Paluch-Shimon S., Huober J., Park I.H., Tredan O., Chen S.C., Manso L. (2017). MONARCH 3: Abemaciclib as initial therapy for advanced breast cancer. J. Clin. Oncol..

[92] Slamon D.J., Neven P., Chia S., Fasching P.A., De Laurentiis M., Im S.A., Petrakova K., Bianchi G.V., Esteva F.J., Martin M. (2018). Phase III randomized study of ribociclib and fulvestrant in hormone receptor-positive, human epidermal growth factor receptor 2-negative advanced breast cancer: MONALEESA-3. J. Clin. Oncol..

[93] Dulos J., Carven G.J., van Boxtel S.J., Evers S., Driessen-Engels L.J., Hobo W., Gorecka M.A., de Haan A.F., Mulders P., Punt C.J. (2012). PD-1 blockade augments Th1 and Th17 and suppresses Th2 responses in peripheral blood from patients with prostate and advanced melanoma cancer. J. Immunother..

[94] Yoshino K., Nakayama T., Ito A., Sato E., Kitano S. (2019). Severe colitis after PD-1 blockade with nivolumab in advanced melanoma patients: Potential role of Th1-dominant immune response in immune-related adverse events: Two case reports. BMC Cancer.

[95] Tanaka R., Okiyama N., Okune M., Ishitsuka Y., Watanabe R., Furuta J., Ohtsuka M., Otsuka A., Maruyama H., Fujisawa Y. (2017). Serum level of interleukin-6 is increased in nivolumab-associated psoriasiform dermatitis and tumor necrosis factor-alpha is a biomarker of nivolumab recativity. J. Dermatol. Sci..

[96] Tanaka R., Ichimura Y., Kubota N., Saito A., Nakamura Y., Ishitsuka Y., Watanabe R., Fujisawa Y., Kanzaki M., Mizuno S. (2020). Activation of CD8 T cells accelerates anti-PD-1 antibody-induced psoriasis-like dermatitis through IL-6. Commun. Biol..

[97] Tarhini A.A., Zahoor H., Lin Y., Malhotra U., Sander C., Butterfield L.H., Kirkwood J.M. (2015). Baseline circulating IL-17 predicts toxicity while TGF-beta1 and IL-10 are prognostic of relapse in ipilimumab neoadjuvant therapy of melanoma. J. Immunother. Cancer.

[98] Lim S.Y., Lee J.H., Gide T.N., Menzies A.M., Guminski A., Carlino M.S., Breen E.J., Yang J.Y.H., Ghazanfar S., Kefford R.F. (2019). Circulating cytokines predict immune-related toxicity in melanoma patients receiving Anti-PD-1-based immunotherapy. Clin. Cancer Res..

[99] Andrykowski M.A., Holland J.C. (2010). Kangas, M. Posttraumatic stress disorder associated with cancer diagnosis and treatment. Psycho-Oncology.

[100] Kähler K.C., Hassel J.C., Heinzerling L., Loquai C., Thoms K.-M., Ugurel S., Zimmer L., Gutzmer R. (2020). Side effect management during immune checkpoint blockade using CTLA-4 and PD-1 antibodies for metastatic melanoma—An update. J. Dtsch. Dermatol. Ges..

[101] Kangas M., Henry J.L., Bryant R.A. (2002). Posttraumatic stress disorder following cancer: A conceptual and empirical review. Clin. Psychol. Rev..

[102] Temel J.S., Shaw A.T., Greer J.A. (2016). Challenge of prognostic uncertainty in the modern era of cancer therapeutics. J. Clin. Oncol..

[103] Krigel S., Myers J., Befort C., Krebill H., Klemp J. (2014). Cancer changes everything!’ Exploring the lived experiences of women with metastatic breast cancer. Int. J. Palliat. Nurs..

[104] Andrykowski M.A., Steffens R.F., Bush H.M., Tucker T.C. (2015). Lung cancer diagnosis and treatment as a traumatic stressor in DSM-IV and DSM-5: Prevalence and relationship to mental health outcomes. J. Trauma. Stress.

[105] Lillis T.A., Gerhart J., Bouchard L.C., Cvengros J., O’Mahony S., Kopkash K., Kabaker K.B., Burns J. (2018). Sleep disturbance mediates the association of post-traumatic stress disorder symptoms and pain in patients with cancer. Am. J. Hosp. Palliat. Care.

[106] Tevaarwerk A.J., Lee J.-W., Terhaar A., Sesto M.E., Smith M.L., Cleeland C.S., Fisch M.J. (2016). Working after a metastatic cancer diagnosis: Factors affecting employment in the metastatic setting from ECOG-ACRIN’s Symptom Outcomes and Practice Patterns study. Cancer.

[107] Cavanna L., Monfredo M., Citterio C. (2019). Job loss and return to work of patients with cancer. A prospective observational study on 416 cancer patients. Recenti Prog. Med..

[108] Rotter J., Spencer J.C., Wheeler S.B. (2019). Financial toxicity in advanced and metastatic cancer: Overburdened and underprepared. J. Oncol. Pract..

[109] Low C.A. (2015). Stanton, A.L. Activity disruption and depressive symptoms in women living with metastatic breast cancer. Health Psychol..

[110] Cardoso F., Harbeck N., Mertz S., Fenech D. (2016). Evolving psychosocial, emotional, functional, and support needs of women with advanced breast cancer: Results from the Count Us, Know Us, Join Us and Here & Now surveys. Breast.

[111] Maunsell E., Brisson J., Deschênes L. (1995). Social support and survival among women with breast cancer. Cancer.

[112] Koopman C., Hermanson K., Diamond S., Angell K., Spiegel D. (1998). Social support, life stress, pain and emotional adjustment to advanced breast cancer. Psychooncology.

[113] Dinh K.T., Aizer A.A., Muralidhar V., Mahal B.A., Chen Y.W., Beard C.J., Choueiri T.K., Hoffman K.E., Hu J.C., Martin N.E. (2018). Increased vulnerability to poorer cancer-specific outcomes following recent divorce. Am. J. Med..

[114] Pyter L.M., Suarez-Kelly L.P., Carson W.E., Kaur J., Bellisario J., Bever S.R. (2017). Novel rodent model of breast cancer survival with persistent anxiety-like behavior and inflammation. Behav. Brain Res..

[115] Khoja L., Atenafu E.G., Ye Q., Gedye C., Chappell M., Hogg D., Butler M.O., Joshua A.M. (2016). Real-world efficacy, toxicity and clinical management of ipilimumab treatment in metastatic melanoma. Oncol. Lett..

[116] Simeone E., Grimaldi A.M., Esposito A., Curvietto M., Palla M., Paone M., Mozzillo N., Ascierto P.A. (2014). Serious haematological toxicity during and after ipilimumab treatment: A case series. J. Med. Case Rep..

[117] Couey M.A., Bell R.B., Patel A.A., Romba M.C., Crittenden M.R., Curti B.D., Urba W.J., Leidner R.S. (2019). Delayed immune-related events (DIRE) after discontinuation of immunotherapy: Diagnostic hazard of autoimmunity at a distance. J. Immunother. Cancer.

[118] Thanarajasingam G., Atherton P.J., Novotny P.J., Loprinzi C.L., Sloan J.A., Grothey A. (2016). Longitudinal adverse event assessment in oncology clinical trials: The Toxicity over Time (ToxT) analysis of Alliance trials NCCTG N9741 and 979254. Lancet Oncol..

[119] Struzik L., Vermani M., Duffin J., Katzman M.A. (2004). Anxiety sensitivity as a predictor of panic attacks. Psychiatry Res..

[120] Rasic D.T., Belik S.L., Bolton J.M., Chochinov H.M., Sareen J. (2008). Cancer, mental disorders, suicidal ideation and attempts in a large community sample. Psychooncology.

[121] 121. PDQ Supportive and Palliative Care Editorial Board, PDQ Cancer-Related Post-Traumatic Stress. National Cancer Institute: Bethesda, MD, USA. Updated 10/30/2019. https://www.cancer.gov/about-cancer/coping/survivorship/new-normal/ptsd-hp-pdq.

[122] Butler L.D., Koopman C., Classen C., Spiegel D. (1999). Traumatic stress, life events, and emotional support in women with metastatic breast cancer: Cancer-related traumatic stress symptoms associated with past and current stressors. Health Psychol..

[123] Rogiers A., Leys C., Lauwyck J., Schembri A., Awada G., Schwarze J.K., De Cremer J., Theuns P., Maruff P., De Ridder M. (2020). Neurocognitive function, psychosocial outcome, and health-related quality of life of the first-generation metastatic melanoma survivors treated with ipilimumab. J. Immunol. Res..

[124] Rogiers A., Leys C., De Cremer J., Awada G., Schembri A., Theuns P., De Ridder M., Neyns B. (2019). Health-related quality of life, emotional burden, and neurocognitive function in the first generatio of metastatic melanoma survivors treated with pembrolizumab: A longitudinal pilot study. Support. Care Cancer.

[125] Smith S.K., Zimmerman S., Williams C.S., Benecha H., Abernethy A.P., Mayer D.K., Edwards L.J., Ganz P.A. (2011). Post-traumatic stress symptoms in long-term non-Hodgkin’s lymphoma survivors: Does time heal?. J. Clin. Oncol..

[126] Pérez S., Conchado A., Andreu Y., Galdón M.J., Cardeña E., Ibáñez E., Durá E. (2016). Acute stress trajectories 1 year after a breast cancer diagnosis. Support. Care Cancer.

[127] Andersen B.L., Valentine T.R., Lo S.B., Carbone D.P., Presley C.J., Shields P.G. (2020). Newly diagnosed patients with advanced non-small cell lung cancer: A clinical description of those with moderate to severe depressive symptoms. Lung Cancer.

[128] Gibbons A., Groarke A., Sweeney K. (2016). Predicting general and cancer-related distress in women with newly diagnosed breast cancer. BMC Cancer.

[129] Palesh O., Scheiber C., Kesler S., Mustian K., Koopman C., Schapira L. (2018). Management of side effects during and post-treatment in breast cancer survivors. Breast J..

[130] Ebede C.C., Jang Y., Escalante C.P. (2017). Cancer-related fatigue in cancer survivorship. Med. Clin. N. Am..

[131] Mustafa M., Carson-Stevens A., Gillespie D., Edwards A.G. (2013). Psychological interventions for women with metastatic breast cancer. Cochr. Database Syst. Rev..

[132] Hasson-Ohayon I., Goldzweig G., Dorfman C., Uziely B. (2014). Hope and social support utilisation among different age groups of women with breast cancer and their spouses. Psychol. Health.

[133] Vilhauer R.P. (2011). ‘Them’ and ‘us’: The experiences of women with metastatic disease in mixed-stage versus stage-specific breast cancer support groups. Psychol. Health.

[134] Verma V., Sprave T., Haque W., Simone C.B., Chang J.Y., Welsh J.W., Thomas C.R. (2018). A systematic review of the cost and cost-effectiveness studies of immune checkpoint inhibitors. J. Immunother. Cancer.

[135] Diaby V., Tawk R., Sanogo V., Xiao H., Montero A.J. (2015). A review of systematic reviews of the cost-effectiveness of hormone therapy, chemotherapy, and targeted therapy for breast cancer. Breast Cancer Res. Treat..

[136] Zafar S.Y., Peppercorn J.M., Schrag D., Taylor D.H., Goetzinger A.M., Zhong X., Abernethy A.P. (2013). The financial toxicity of cancer treatment: A pilot study assessing out-of-pocket expenses and the insured cancer patient’s experience. Oncologist.

[137] Loscalzo M., Clark K., Pal S., Pirl W.F. (2013). Role of biopsychosocial screening in cancer care. Cancer J..

[138] Aizer A.A., Chen M.H., McCarthy E.P., Mendu M.L., Koo S., Wilhite T.J., Graham P.L., Choueiri T.K., Hoffman K.E., Martin N.E. (2013). Marital status and survival in patients with cancer. J. Clin. Oncol..

[139] Pirl W.F., Fann J.R., Greer J.A., Braun I., Deshields T., Fulcher C., Harvey E., Holland J., Kennedy V., Lazenby M. (2014). Recommendations for the Implementation of Distress Screening Programs in Cancer Centers Report From the American Psychosocial Oncology Society (APOS), Association of Oncology Social Work (AOSW), and Oncology Nursing Society (ONS) Joint Task Force. Cancer.

[140] Penedo F.J., Oswald L.B., Kronenfeld J.P., Garcia S.F., Cella D., Yanez B. (2020). The increasing value of eHealth in the delivery of patient-centred cancer care. Lancet Oncol..

[141] Jim H.S.L., Hoogland A.I., Brownstein N.C., Barata A., Dicker A.P., Knoop H., Gonzalez B.D., Perkins R., Rollison D., Gilbert S.M. (2020). Innovations in research and clinical care using patient-generated health data. CA Cancer J. Clin..

[142] Basch E., Deal A.M., Kris M.G., Scher H.I., Hudis C.A., Sabbatini P., Rogak L., Bennett A.V., Dueck A.C., Atkinson T.M. (2016). Symptom Monitoring With Patient-Reported Outcomes During Routine Cancer Treatment: A Randomized Controlled Trial. J. Clin. Oncol..

[143] Basch E., Deal A.M., Dueck A.C., Scher H.I., Kris M.G., Hudis C., Schrag D. (2017). Overall survival results of a trial assessing patient-reported outcomes for symptom monitoring during routine cancer treatment. JAMA.

[144] Denis F., Lethrosne C., Pourel N., Molinier O., Pointreau Y., Domont J., Bourgeois H., Senellart H., Tremolieres P., Lizee T. (2017). Randomized Trial Comparing a Web-Mediated Follow-up With Routine Surveillance in Lung Cancer Patients. J. Natl. Cancer Inst..

[145] Yanez B., Bouchard L.C., Cella D., Sosman J.A., Kircher S.M., Mohindra N.A., Cristofanilli M., Penedo F.J. (2019). Patient-centered engagement and symptom/toxicity monitoring in the new era of tumor next-generation sequencing and immunotherapy: The OncoTool and OncoPRO platforms. Cancer.

[146] Wagner L.I., Schink J., Bass M., Patel S., Diaz M.V., Rothrock N., Pearman T., Gershon R., Penedo F.J., Rosen S. (2015). Bringing PROMIS to practice: Brief and precise symptom screening in ambulatory cancer care. Cancer.

[147] Garcia S.F., Wortman K., Cella D., Wagner L.I., Bass M., Kircher S., Pearman T., Penedo F.J. (2019). Implementing electronic health record-integrated screening of patient-reported symptoms and supportive care needs in a comprehensive cancer center. Cancer.

[148] Chung A., Stover A.M., Wagner L.I., LeBlanc T.W., Topalaglu U., Zafar Y., Zullig L.L., Smeltzer P., Basch E.M. (2017). Harmonization of patient-reported outcomes into EHRs at four cancer hospital outpatient clinics for patient care and quality assessment. J. Clin. Oncol..

[149] Teo I., Krishnan A., Lee G.L. (2019). Psychosocial interventions for advanced cancer patients: A systematic review. Psychooncology.

[150] Clayton J.M., Butow P.N., Tattersall M.H., Devine R.J., Simpson J.M., Aggarwal G., Clark K.J., Currow D.C., Elliott L.M., Lacey J. (2007). Randomized controlled trial of a prompt list to help advanced cancer patients and their caregivers to ask questions about prognosis and end-of-life care. J. Clin. Oncol..

[151] Epstein R.M., Duberstein P.R., Fenton J.J., Fiscella K., Hoerger M., Tancredi D.J., Xing G., Gramling R., Mohile S., Franks P. (2017). Effect of a Patient-Centered Communication Intervention on Oncologist-Patient Communication, Quality of Life, and Health Care Utilization in Advanced Cancer: The VOICE Randomized Clinical Trial. JAMA Oncol..

[152] Shirai Y., Fujimori M., Ogawa A., Yamada Y., Nishiwaki Y., Ohtsu A., Uchitomi Y. (2012). Patients’ perception of the usefulness of a question prompt sheet for advanced cancer patients when deciding the initial treatment: A randomized, controlled trial. Psycho-Oncol..

[153] Meropol N.J., Egleston B.L., Buzaglo J.S., Balshem A., Benson A.B., Cegala D.J., Cohen R.B., Collins M., Diefenbach M.A., Miller S.M. (2013). A Web-based communication aid for patients with cancer: The CONNECT Study. Cancer.

[154] Walczak A., Butow P.N., Tattersall M.H., Davidson P.M., Young J., Epstein R.M., Costa D.S., Clayton J.M. (2017). Encouraging early discussion of life expectancy and end-of-life care: A randomised controlled trial of a nurse-led communication support program for patients and caregivers. Int. J. Nurs. Stud..

[155] El-Jawahri A., Podgurski L.M., Eichler A.F., Plotkin S.R., Temel J.S., Mitchell S.L., Chang Y.C., Barry M.J., Volandes A.E. (2010). Use of Video to Facilitate End-of-Life Discussions With Patients With Cancer: A Randomized Controlled Trial. J. Clin. Oncol..

[156] Leighl N.B., Shepherd H.L., Butow P.N., Clarke S.J., McJannett M., Beale P.J., Wilcken N.R., Moore M.J., Chen E.X., Goldstein D. (2011). Supporting treatment decision making in advanced cancer: A randomized trial of a decision aid for patients with advanced colorectal cancer considering chemotherapy. J. Clin. Oncol..

[157] Kim H.S., Shin S.J., Kim S.C., An S., Rha S.Y., Ahn J.B., Cho B.C., Choi H.J., Sohn J.H., Kim H.S. (2013). Randomized controlled trial of standardized education and telemonitoring for pain in outpatients with advanced solid tumors. Support. Care Cancer.

[158] Kaasa S., Loge J.H., Aapro M., Albreht T., Anderson R., Bruera E., Brunelli C., Caraceni A., Cervantes A., Currow D.C. (2018). Integration of oncology and palliative care: A Lancet Oncology Commission. Lancet Oncol..

[159] Vanbutsele G., Pardon K., Van Belle S., Surmont V., De Laat M., Colman R., Eecloo K., Cocquyt V., Geboes K., Deliens L. (2018). Effect of early and systematic integration of palliative care in patients with advanced cancer: A randomised controlled trial. Lancet Oncol..

[160] Ferrell B.R., Temel J.S., Temin S., Alesi E.R., Balboni T.A., Basch E.M., Firn J.I., Paice J.A., Peppercorn J.M., Phillips T. (2017). Integration of palliative care into standard oncology care: American Society of Clinical Oncology clinical practice guideline update. J. Clin. Oncol..

[161] National Comprehensive Cancer Network, NCCN Clinical Practice Guidelines in Oncology (NCCN Guidelines^®^) Palliative Care. Version 1.2020. Updated 020/7/2020. https://www.nccn.org/professionals/physician_gls/pdf/palliative.pdf.

[162] Griffiths R.R., Johnson M.W., Carducci M.A., Umbricht A., Richards W.A., Richards B.D., Cosimano M.P., Klinedinst M.A. (2016). Psilocybin produces substantial and sustained decreases in depression and anxiety in patients with life-threatening cancer: A randomized double-blind trial. J. Psychopharmacol..

[163] Good P., Haywood A., Gogna G., Martin J., Yates P., Greer R., Hardy J. (2019). Oral medicinal cannabinoids to relieve symptom burden in the palliative care of patients with advanced cancer: A double-blind, placebo controlled, randomised clinical trial of efficacy and safety of cannabidiol (CBD). BMC Palliat. Care.

[164] Greer J.A., Traeger L., Bemis H., Solis J., Hendriksen E.S., Park E.R., Pirl W.F., Temel J.S., Prigerson H.G., Safren S.A. (2012). A pilot randomized controlled trial of brief cognitive-behavioral therapy for anxiety in patients with terminal cancer. Oncologist.

[165] Badr H., Smith C.B., Goldstein N.E., Gomez J.E., Redd W.H. (2015). Dyadic psychosocial intervention for advanced lung cancer patients and their family caregivers: Results of a randomized pilot trial. Cancer.

[166] Yanez B., McGinty H.L., Mohr D.C., Begale M.J., Dahn J.R., Flury S.C., Perry K.T., Penedo F.J. (2015). Feasibility, acceptability, and preliminary efficacy of a technology-assisted psychosocial intervention for racially diverse men with advanced prostate cancer. Cancer.

[167] Chambers S.K., Occhipinti S., Foley E., Clutton S., Legg M., Berry M., Stockler M.R., Frydenberg M., Gardiner R.A., Lepore S.J. (2017). Mindfulness-Based Cognitive Therapy in Advanced Prostate Cancer: A Randomized Controlled Trial. J. Clin. Oncol..

[168] Cheung E.O., Cohn M.A., Dunn L.B., Melisko M.E., Morgan S., Penedo F.J., Salsman J.M., Shumay D.M., Moskowitz J.T. (2017). A randomized pilot trial of a positive affect skill intervention (lessons in linking affect and coping) for women with metastatic breast cancer. Psychooncology.

[169] Do Carmo T.M., Paiva B.S.R., de Oliveira C.Z., Nascimento M.S.A., Paiva C.E. (2017). The feasibility and benefit of a brief psychosocial intervention in addition to early palliative care in patients with advanced cancer to reduce depressive symptoms: A pilot randomized controlled clinical trial. BMC Cancer.

[170] Lapid M.I., Rummans T.A., Brown P.D., Frost M.H., Johnson M.E., Huschka M.M., Sloan J.A., Richardson J.W., Hanson J.M., Clark M.M. (2007). Improving the quality of life of geriatric cancer patients with a structured multidisciplinary intervention: A randomized controlled trial. Palliat. Support. Care.

[171] Clark M.M., Rummans T.A., Atherton P.J., Cheville A.L., Johnson M.E., Frost M.H., Miller J.J., Sloan J.A., Graszer K.M., Haas J.G. (2013). Randomized controlled trial of maintaining quality of life during radiotherapy for advanced cancer. Cancer.

[172] Gaston-Johansson F., Fall-Dickson J.M., Nanda J.P., Sarenmalm E.K., Browall M., Goldstein N. (2013). Long-term effect of the self-management comprehensive coping strategy program on quality of life in patients with breast cancer treated with high-dose chemotherapy. Psychooncology.

[173] Northouse L.L., Mood D.W., Schafenacker A., Kalemkerian G., Zalupski M., LoRusso P., Hayes D.F., Hussain M., Ruckdeschel J., Fendrick A.M. (2013). Randomized clinical trial of a brief and extensive dyadic intervention for advanced cancer patients and their family caregivers. Psychooncology.

[174] Mosher C.E., Secinti E., Johns S.A., O’Neil B.H., Helft P.R., Shahda S., Jalal S.I., Champion V.L. (2018). Examining the effect of peer helping in a coping skills intervention: A randomized controlled trial for advanced gastrointestinal cancer patients and their family caregivers. Qual. Life Res..

[175] Penedo F.J., Fox R.S., Oswald L.B., Moreno P.I., Boland C.L., Estabrook R., McGinty H.L., Mohr D.C., Begale M.J., Dahn J.R. (2020). Technology-Based Psychosocial Intervention to Improve Quality of Life and Reduce Symptom Burden in Men with Advanced Prostate Cancer: Results from a Randomized Controlled Trial. Int. J. Behav. Med..

[176] Ward S., Donovan H., Gunnarsdottir S., Serlin R.C., Shapiro G.R., Hughes S. (2008). A randomized trial of a representational intervention to decrease cancer pain (RIDcancerPain). Health Psychol..

[177] Chan C.W., Richardson A., Richardson J. (2011). Managing symptoms in patients with advanced lung cancer during radiotherapy: Results of a psychoeducational randomized controlled trial. J. Pain Symptom Manag..

[178] Kwekkeboom K.L., Abbott-Anderson K., Cherwin C., Roiland R., Serlin R.C., Ward S.E. (2012). Pilot randomized controlled trial of a patient-controlled cognitive-behavioral intervention for the pain, fatigue, and sleep disturbance symptom cluster in cancer. J. Pain Symptom Manag..

[179] Ducloux D., Guisado H., Pautex S. (2013). Promoting sleep for hospitalized patients with advanced cancer with relaxation therapy: Experience of a randomized study. Am. J. Hosp. Palliat. Care.

[180] Farquhar M.C., Prevost A.T., McCrone P., Brafman-Price B., Bentley A., Higginson I.J., Todd C., Booth S. (2014). Is a specialist breathlessness service more effective and cost-effective for patients with advanced cancer and their carers than standard care? Findings of a mixed-method randomised controlled trial. BMC Med..

[181] Alcalde-Castro M.J., Soto-Perez-de-Celis E., Covarrubias-Gómez A., Sánchez-Román S., Quiróz-Friedman P., Navarro-Lara Á., Ramos-Lopez W.A., Moreno-García M.L., Contreras-Garduño S., Perez-Montessoro V. (2020). Symptom assessment and early access to supportive and palliative care for patients with advanced solid tumors in Mexico. J. Palliat. Care.

[182] Wright A.A., Zhang B., Ray A., Mack J.W., Trice E., Balboni T., Mitchell S.L., Jackson V.A., Block S.D., Maciejewski P.K. (2008). Associations between end-of-life discussions, patient mental health, medical care near death, and caregiver bereavement adjustment. JAMA.

[183] Prater L.C., Wickizer T., Bower J.K., Bose-Brill S. (2019). The impact of advance care planning on end-of-life care: Do the type and timing make a difference for patients with advanced cancer referred to hospice?. Am. J. Hosp. Palliat. Care.

[184] Emiloju O.E., Djibo D.A.M., Ford J.G. (2020). Association between the timing of goals-of-care discussion and hospitalization outcomes in patients with metastatic cancer. Am. J. Hosp. Palliat. Care.

[185] Starr L.T., Ulrich C.M., Corey K.L., Meghani S.H. (2019). Associations among end-of-life discussions, health-care utilization, and costs in persons with advanced cancer: A systematic review. Am. J. Hosp. Palliat. Care.

[186] Agarwal R. (2018). Epstein, A.S. Advance care planning and end-of-life decision making for patients with cancer. Semin. Oncol. Nurs..

[187] LoPresti M.A., Dement F., Gold H.T. (2016). End-of-life care for people with cancer from ethnic minority groups: A systematic review. Am. J. Hosp. Palliat. Care..

[188] Choudry M., Latif A., Warburton K.G. (2018). An overview of the spiritual importances of end-of-life care among the five major faiths of the United Kingdom. Clin. Med. (Lond.).

[189] PDQ Supportive and Palliative Care Editorial Board, PDQ Planning the Transition to End-of-Life Care in Advanced Cancer. Bethesda, MD: National Cancer Institute. Updated 06/25/2020. https://www.cancer.gov/about-cancer/advanced-cancer/planning/end-of-life-hp-pdq.

[190] Duncan M., Moschopoulou E., Herrington E., Deane J., Roylance R., Jones L., Bourke L., Morgan A., Chalder T., Thaha M.A. (2017). Review of systematic reviews of non-pharmacological interventions to improve quality of life in cancer survivors. BMJ Open.

[191] Baguley B.J., Bolam K.A., Wright O.R.L., Skinner T.L. (2017). The effect of nutrition therapy and exercise on cancer-related fatigue and quality of life in men with prostate cancer: A systematic review. Nutrients.

[192] Frankl V., Lasch I. (1992). Man’s Search for Meaning: An Introduction to Logotherapy.

[193] Montross Thomas L.P., Meier E.A., Irwin S.A. (2014). Meaning-centered psychotherapy: A form of psychotherapy for patients with cancer. Curr. Psychiatry Rep..

[194] Hyland K., Eisel S., Hoogland A., Nelson A., Knoop H., Jacobsen P., Jim H. (2020). Perpetuating and exacerbating factors as mediators of change in a CBT-based intervention for fatigue in patients with chronic myeloid leukemia. Psychooncology.

[195] Buckley J. (2004). Herth, K. Fostering hope in terminally ill patients. Nurs. Stand..

[196] Esbensen B.A., Thomsen T. (2011). Quality of life and hope in elderly people with cancer. Open J. Nurs..

[197] Sanatani M., Schreier G., Stitt L. (2008). Level and direction of hope in cancer patients: An exploratory longitudinal study. Support. Care Cancer.

[198] Gum A., Snyder C.R. (2002). Coping with terminal illness: The role of hopeful thinking. J. Palliat. Med..

[199] Leary M.R., Tate E.B., Adams C.E., Allen A.B., Hancock J. (2007). Self-compassion and reactions to unpleasant self-relevant events: The implications of treating oneself kindly. J. Personal. Soc. Psychol..

[200] Diedrich A., Burger J., Kirchner M., Berking M. (2017). Adaptive emotion regulation mediates the relationship between self-compassion and depression in individuals with unipolar depression. Psychol. Psychother..

[201] Sydenham M., Beardwood J., Rimes K.A. (2017). Beliefs about emotions, depression, anxiety and fatigue: A mediational analysis. Behav. Cogn. Psychother..

[202] Alda M., Puebla-Guedea M., Rodero B., Demarzo M., Montero-Marin J., Roca M., Garcia-Campayo J. (2016). Zen meditation, length of telomeres, and the role of experiential avoidance and compassion. Mindfulness (NY).

[203] Larson A.G., Morris K.J., Juckett M.B., Coe C.L., Broman A.T., Costanzo E.S. (2019). Mindfulness, experiential avoidance, and recovery from hematopoietic stem cell transplantation. Ann. Behav. Med..

[204] Lo C., Hales S., Chiu A., Panday T., Malfitano C., Jung J., Rydall A., Li M., Nissim R., Zimmermann C. (2019). Managing cancer and living meaningfully (CALM): Randomised feasibility trial in patients with advanced cancer. BMJ Support. Palliat. Care.

[205] Rodin G., An E., Shnall J., Malfitano C. (2020). Psychological interventions for patients with advanced disease: Implications for oncology and palliative care. J. Clin. Oncol..

